# Anhydrobiosis as a Model of Aging and Longevity: The Role of Autophagy and Metabolism in Yeast Cells

**DOI:** 10.1007/s00248-026-02782-7

**Published:** 2026-04-28

**Authors:** Naga Pavan Kumar Reddy Jonnagiri, Marek Kieliszek

**Affiliations:** Department of Food Biotechnology and Microbiology, Institute of Food Sciences, Warsaw University of Life Sciences—SGGW, Nowoursynowska 159 C, Warsaw, 02-776 Poland

**Keywords:** Anhydrobiosis, Yeast, Autophagy, Aging, Longevity

## Abstract

Anhydrobiosis, the ability of cells to endure severe dehydration, provides a valuable model for investigating cellular resilience, aging, and long-term endurance processes. Yeast species, such as *Saccharomyces cerevisiae* and non-conventional yeasts, are valuable tools for understanding how stress-response pathways, metabolic regulation, and proteostasis contribute to desiccation tolerance. Experimental studies show that trehalose build-up, High Osmolarity Glycerol (HOG)-MAPK signalling, mitochondrial energy maintenance, and protein quality control networks are all protective of cellular structures during water loss and facilitate the rapid recovery of cellular structures upon rehydration. In yeast, these processes are tightly linked to chronological lifespan regulation and stress resistance in non-dividing cells. Multi-omics studies, such as transcriptomics, lipidomics, and chromatin accessibility studies, indicate widespread remodelling of metabolic pathways, membrane architecture, and chromatin states, with transcriptional stress memory maintained by epigenetic mechanisms to improve survival during repeated cycles of dehydration. Anhydrobiosis has been applied in biotechnology, agriculture, and biomedical science, with yeast-based systems being used to produce active dry yeast, preserve enzymes and vaccines, and create stress-tolerant microbial inoculants to enhance crop growth. Yeast anhydrobiosis integrates genetics, biophysics, and systems biology to explain cell survival under extreme stress and to guide the engineering of more stable biological systems. Overall, this summary of recent findings on genetic determinants, molecular pathways, and yeast responses to stress during anhydrobiosis provides valuable insights into aging and resilience mechanisms and establishes anhydrobiosis as a conceptual framework for studying conserved cellular maintenance strategies rather than a direct model of organismal longevity.

## Introduction

Anhydrobiosis is a state in which an organism survives extreme drying and almost complete water loss [1]. It plays a crucial role in understanding how aging and longevity occur at the molecular level [2]. It has been found in animals such as tardigrades, nematodes, and yeast. It is worth emphasizing that this process involves significant changes in cells that enable them to survive for many years. Furthermore, their metabolism is completely shut down [3, 4]. These adaptations share numerous similarities with processes that help slow down the aging process, including autophagy, proteostasis, and metabolic reprogramming. Just as dietary restriction, is known to help organisms live longer, triggering anhydrobiosis activates stress-response systems that protect cells from damage caused by oxidation, protein clumping, and low energy [5–8]. The chronological lifespan (CLS) model is a standard method for assessing longevity in yeast. It measures the survival of non-dividing cells over time, combining essential aging-related parameters such as stress resistance, metabolic state, and cellular maintenance capacity [9].

The ability to study the phenomenon of anhydrobiosis in a simple organism like *S. cerevisiae* provides an excellent opportunity to examine these connections in more detail, as the process of drying out of yeast has major longevity pathways in common with more complex organisms, such as mammals [10]. The close interconnection between proteostasis and metabolic homeostasis is crucial for survival in the absence of water [11]. Dehydration activates autophagy, a process that helps remove damaged cellular components and misfolded proteins. Furthermore, such processes prevent the accumulation of harmful aggregates, which consequently has a significant impact on cell viability [12, 13]. Trehalose helps maintain the structure of membranes and proteins in a dry state, while glycogen provides cells with the energy necessary for recovery after hydration [11, 14]. Together, these responses indicate that dehydrated cells actively limit damage to preserve long-term functionality. Table 1 summarizes the key mechanisms linking anhydrobiosis to longevity in yeast.

**Table 1:** Core mechanisms linking anhydrobiosis to longevity in yeast

Mechanism	Role in Anhydrobiosis	Role in Longevity	Key Regulators/Effectors
Autophagy	Clearance of damaged organelles/proteins during dehydration stress; prevents lethal aggregate formation [11]	Suppresses cellular senescence by recycling damaged components; removes toxic aggregates [15]	Atg proteins, TORC1 pathway [16]
Proteostasis	Enhanced chaperone activity (folding), proteasomal/autophagic degradation prevent desiccation-induced aggregation, denaturation [17]	Maintains protein integrity during aging; delays functional decline [18]	Hsp, Ubiquitin Proteasome System [18]
Metabolic shifts	Accumulation of trehalose, glycerol [19]	Mimics dietary restriction (energy sensing/signalling); enhances stress resistance; preserves energy homeostasis [20]	Snf1 (AMPK ortholog), Msn2/4, Tps1 (trehalose-6-P synthase), PKA [21]
Epigenetic memory	Stress-induced chromatin remodeling primes rapid reactivation of stress-response genes [22]	Promotes adaptive responses to recurrent stress, contributing to cellular longevity [23]	Histone acetylation,/deacetylation, methylation [23]

Dehydration stress activates conserved osmotic stress-responsive signal pathways that facilitate cellular adaptation and survival. These pathways help activate genes involved in the production of compatible solutes, including trehalose and glycerol, which are essential for cell survival under low-water conditions [19, 24–26]. In this review, the terms dehydration and desiccation refer to progressive and extreme water loss, respectively, and anhydrobiosis is the reversible physiological condition that allows survival under these conditions. Importantly, long-term dehydration adaptation is not limited to acute stress signaling. The epigenetic regulation of histone acetylation, methylation, and chromatin remodeling forms a memory of stress that enhances survival during repeated dehydration–rehydration cycles [27]. Such chromatin alterations enable faster transcriptional responses to recurrent stress, revealing molecular stress memory in single-celled organisms. Similar mechanisms in plants and other eukaryotes highlight the broader relevance of yeast stress biology to resilience and longevity [28, 29].

This review summarizes current evidence on the genetic factors required for yeast survival under desiccation. Key stress-responsive signaling pathways and their impact on gene expression during extreme water stress are discussed [30]. Recent reports that epigenetic regulation mediates stress memory and desiccation resistance are also taken into consideration [31]. It is worth emphasizing that mechanisms associated with anhydrobiosis, such as autophagy and proteostasis, resemble pathways that maintain neuronal function in mammals [32]. Therefore, in this review, we examine how studies on yeast anhydrobiosis may provide conceptual insights into counteracting proteostasis disruption in Alzheimer’s and Parkinson’s disease [33, 34]. Through these connections, yeast emerges not only as a model for desiccation survival but also as a simplified and indirect platform for studying conserved cellular aspects of aging and proteostasis, rather than a direct model of neuroprotection or organismal longevity across life forms. Previous reviews have mainly focused on the structural and physiological aspects of yeast anhydrobiosis. The current review is particularly focused on anhydrobiosis as an aging and longevity model with a special focus on chronological lifespan, autophagy, proteostasis and epigenetic stress memory. This conceptual focus distinguishes the present work from earlier summaries. 

### Longevity Mechanisms Triggered by Dehydration and Dietary Restriction in Yeast Dietary Restriction Induced Longevity Mechanisms

Dietary restriction (DR) extends the chronological lifespan (CLS) of yeast by engaging conserved stress-response and maintenance pathways, which are associated with nutrient sensing, metabolism, and proteostasis [35, 36]. In yeast, CLS reflects the ability of non-dividing, stationary-phase cells to remain viable over time, and serves as a well-established model of post-mitotic aging [37]. Decline in CLS is associated with increased reactive oxygen species (ROS), mitochondrial dysfunction, impaired autophagy, and reduced protein quality control, making it particularly suitable for studying longevity under nutrient limitation [38, 39]. Similar methods include boosting autophagy and enhancing the regulation of protein activity. In DR, TORC1 is inhibited by nutrient deficiency (especially nitrogen or carbon). It then activates Snf1 (yeast AMPK), which initiates autophagy, breaking down cellular components and maintaining energy homeostasis. This process is essential for extending lifespan [40–42]. Protein kinase A (PKA), the central carbon-responsive signaling pathway in yeast, acts as a major upstream regulator of Snf1 activity; reduced glucose availability lowers PKA signalling, thereby relieving inhibition of Snf1 and enabling stress resistance and lifespan extension [43–45]. TORC1 inhibition also reduces ribosomal protein synthesis and enhances the expression of stress-responsive transcription factors (Msn2/4, Gis1, and Hsf1), as well as increases the expression of antioxidant enzymes (Sod2 and catalase), in response to oxidative damage during aging [38, 46–48]. Activation of Snf1 kinase plays a crucial role in regulating cellular metabolism. Its activity helps maintain normal NAD+ levels, which are essential for many metabolic processes and oxidation-reduction reactions [49, 50]. Simultaneously, Snf1 stimulates the formation of new, functional organelles responsible for energy production. In this way, it links metabolic signaling with the maintenance of redox balance and protein quality control (proteostasis). Under conditions of dietary restriction (DR), mitophagy, the removal of damaged or ineffective mitochondria, is enhanced. This mechanism allows the cell to maintain a functional respiratory system while limiting excessive production of reactive oxygen species (ROS) [51, 52]. These processes improve the overall condition of the cell and its ability to survive under stress. It is worth noting that dietary restriction influences chromatin silencing via sirtuin-dependent pathways (Sir2 and Hst1). The activity of these factors stabilizes rDNA regions, reduces genomic instability, and slows the aging process [53–56].

### Anhydrobiosis Induced Longevity Mechanisms

Unlike DR, yeast survival and longevity undergo anhydrobiosis through rapid activation of protective mechanisms that enable cells to endure severe dehydration. Dehydration activates autophagy, often via the HOG pathway, to break down proteins and organelles damaged during drying and to prevent the formation of toxic aggregates during rehydration [11]. HOG1 activation of the HOG-MAPK cascade causes phosphorylation and nuclear retention of stress-responsive transcription factors, especially Msn2/4. This signal triggers osmoadaptive genes such as TPS1 and GPD1, leading to the production of trehalose and glycerol [30, 57, 58].

These metabolites are chemical chaperones and osmolytes that stabilize proteins and membranes and facilitate cytoplasmic vitrification during water loss [33, 59, 60]. Anhydrobiosis is further characterized by transient suppression of translation and the formation of stress granules and processing bodies (P-bodies), which preserve mRNAs for efficient recovery following rehydration [61]. Autophagy is reactivated during rehydration to resume normal metabolism and preserve proteostasis throughout the dehydration cycles [42]. The frequent cycles of dehydration and rehydration are linked to epigenetic changes, such as H3K4 and H3K36 methylation, enabling faster activation of stress-responsive genes and creating a form of transcriptional stress memory [22, 62, 63]. Dietary restriction and anhydrobiosis both extend yeast lifespan by enhancing autophagy, protein stability, and stress resistance. Dietary restriction achieves this by slowing metabolic and mitochondrial adaptations, whereas anhydrobiosis depends on rapid protective mechanisms that help cells endure dehydration. These differences show how shared longevity pathways adapt to different environmental stresses.

## Autophagy and Proteostasis in Anhydrobiosis

**Fig. 1:**
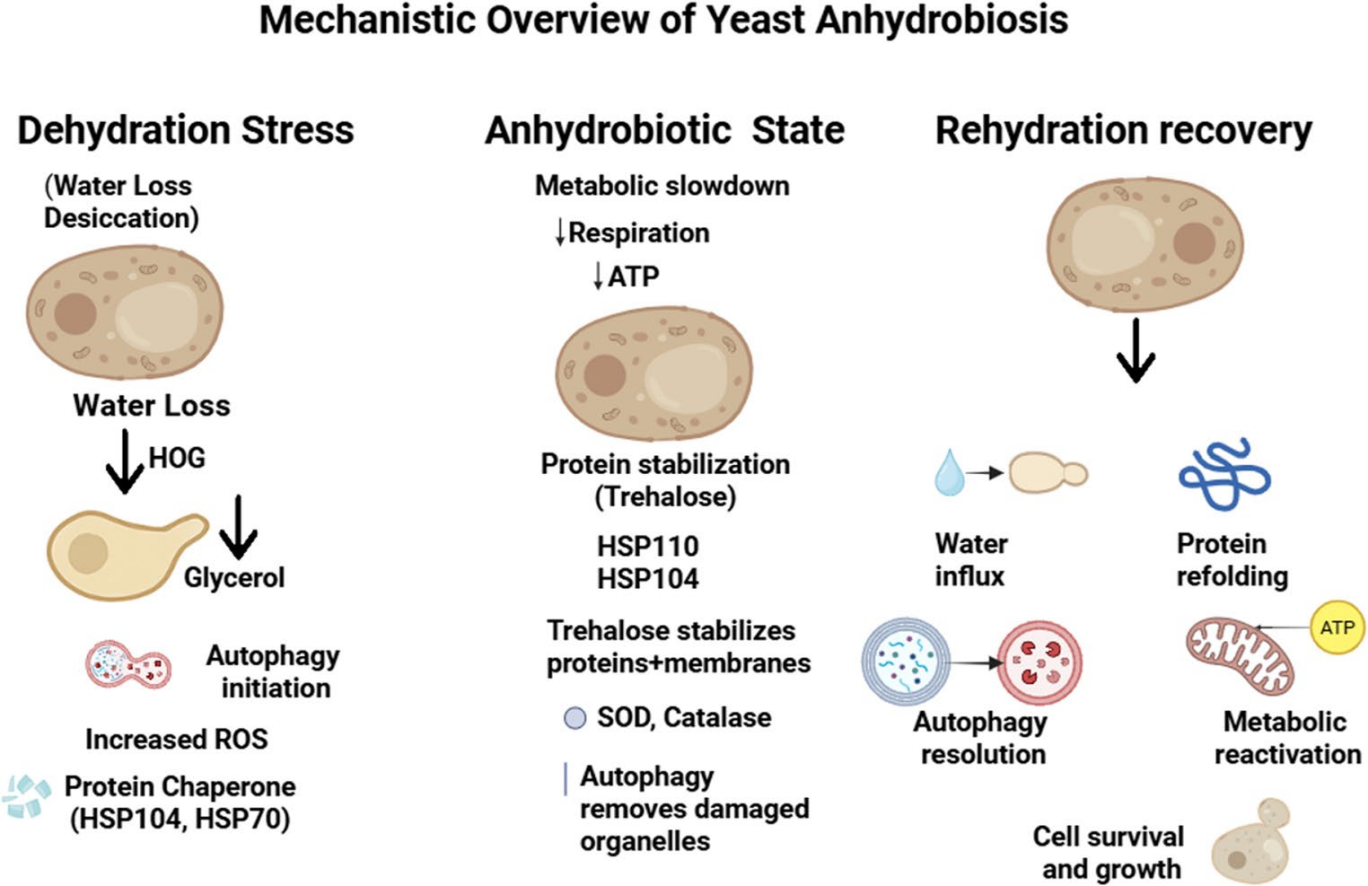
Yeast cell response to dehydration stress. The process involves activating protective pathways, inducing glycerol synthesis, activating autophagy, and inducing heat shock proteins. Under anhydrobiotic conditions, yeast metabolism slows, and trehalose and molecular chaperones are utilized to stabilize proteins and cell membranes. The rehydration process involves the influx of water, protein refolding, cessation of autophagy, and reactivation of metabolism, allowing the cell to regain its growth potential. Figure was prepared with BioRender online software (www.Biorender.com)

Yeast can survive anhydrobiosis because its protein maintenance system reorganizes, and selective autophagy plays a larger role [11]. During desiccation stress, molecular chaperones such as Hsp40 and Hsp104 interact with intrinsically disordered proteins (IDPs) to form biomolecular condensates through liquid–liquid phase separation (LLPS) [64]. Under stressful conditions, cells form specialized aggregates (condensates) that sequester misfolded proteins, preventing their clumping. Once hydrated, the proteins can quickly return to their standard form. It’s worth noting that trehalose regulates the fluidity and reversibility of these aggregates [65]. Furthermore, these condensates function independently of ATP, which allows them to maintain activity even under conditions of complete metabolic inhibition caused by profound dehydration [66]. Trehalose in the yeast cell cytosol participates in the formation and stabilization of reversible protein assemblies. Once the yeast cell is hydrated, the breakdown of protein aggregates is facilitated by chaperone molecules such as Hsp104 and Hsp70, restoring balance in the protein quality control system [67]. Together, these mechanisms demonstrate how yeast reorganizes its proteostatic systems to survive extreme desiccation (Fig. 1).

Meanwhile, certain forms of autophagy precisely regulate cellular resources. Ribophagy degrades 60 S ribosomal subunits to salvage nucleotides in times of nitrogen deprivation, while mitophagy eliminates mitochondria that generate ROS in response to oxidative stress during dehydration [68, 69]. These selective degradation mechanisms maintain proteome integrity and help in the long-term survival of cells under extreme stress conditions [70]. Furthermore, the Atg1, Atg13, and Atg17 complex is activated under conditions of limited nutrient availability or stress factors by inhibiting the TORC1 pathway, triggering the initiation of autophagy in yeast [71]. Cargo-specific receptors regulate selective cellular degradation. Atg32 identifies mitochondrial impairment and triggers mitophagy, while endoplasmic reticulum turnover during ER-phagy is facilitated by the Atg39 and Atg40 receptors [72, 73]. Specific ubiquitination is an initial regulatory step in stress-induced degradation pathways. The E3 ligase Rsp5 attaches damaged proteins to K63-linked ubiquitin chains to facilitate selective autophagy rather than proteasomal degradation during dehydration [74, 75].

During cellular stress responses, K63 polyubiquitination serves as a cargo-recognition signal in selective autophagy rather than as a signal for proteasomal degradation [76, 77]. Additionally, post-translational modifications precisely regulate these processes. O-GlcNAcylation increases proteasome activity, while Atg1 phosphorylation by Hog1 stimulates autophagy under osmotic stress conditions [78, 79]. Post translational modifications also influence intrinsically disordered regions within phase separated condensates maintaining their liquid like and reversible properties and preventing pathological solidification during prolonged stress [80]. A combination of all these processes indicates that yeast transforms and changes proteostasis to an active and organized form of protection against protein damage, a phenomenon also observed in anhydrobiotic tardigrades and nematodes, with significant advantages for aging cells.

Physiological cell activity during dehydration is maintained through the regulation of autophagy and chaperone activity. These processes ensure long-term protein stability. The activity of these pathways limits proteostatic decay in aging yeast populations while simultaneously extending their lifespan. These effects suggest a direct link between lifespan and cells’ ability to survive dehydration [81]. It is noteworthy that these mechanisms demonstrate how cells can maintain their function under harsh environmental conditions and ecological stress.

## Experimental Insights into Yeast Dehydration and Rehydration Mechanisms

Genetic research has shown that desiccation tolerance in yeast is not a single factor but rather determined by numerous protective mechanisms. In a study by Calahan et al. [82], they found that yeast mutants possessing a set of stress response genes (including *TPS1*,* TPS2*,* HSP12*, and *HSP104*) still retained the enhanced desiccation tolerance characteristic of wild-type strains. These results indicate that classical cellular stress response pathways are not essential for survival under extreme dehydration conditions. As reported by Sęk et al. [11], stress conditions (dehydration) increase the expression of genes involved in glycerol synthesis and protective chaperone proteins (e.g., HSPs) in yeast. It is worth emphasizing that yeast has evolved several mechanisms to support cell survival under such unfavorable environmental conditions. Experimental studies have been focusing on trehalose. According to Ratnakumar and Tunnacliffe [83], manipulating of trehalose alone does not completely restore tolerance. Still, Tapia and Koshland [19] showed that trehalose acts as a long-lived molecular chaperone stabilizing proteins during drying. Tapia et al. [84] also demonstrated that artificially increasing intracellular trehalose can confer desiccation tolerance even in yeast cells that are usually sensitive to desiccation. These results indicate that trehalose is used in conjunction with other protective mechanisms to stabilize proteins and membranes in the event of excessive water loss [85]. Key experimental findings on yeast desiccation tolerance are summarized in Table 2.

**Table 2:** Experimental insights into yeast dehydration and rehydration mechanisms

Organism	Main Focus	Key Findings Related to Dehydration/Desiccation Tolerance	References
*S. cerevisiae*	Stress-response gene deletions	Mutants lacking TPS1, TPS2, HSP12, and HSP104 retain wild-type desiccation tolerance, indicating classical stress pathways are not essential.	[82]
*S. cerevisiae*	Stress response and structural adaptations	Stress-signaling and HSP genes are upregulated, while membrane and cell wall remodeling reduce dehydration damage.	[11]
*S. cerevisiae*	Role of trehalose	Manipulation of trehalose alone does not fully restore tolerance, requires combination with other mechanisms	[83]
*S. cerevisiae*	Trehalose as chaperone	Trehalose functions as a long-lived molecular chaperone stabilizing proteins during drying	[19]
*S. cerevisiae*	Artificial trehalose accumulation	Artificially increasing trehalose levels can make normally sensitive cells desiccation tolerant	[84]
*S. cerevisiae*	Glycerol metabolism enzymes	Phosphorylation of Gpd1/Gpd2 defines their stress-specific activity; Gpd1 crucial for hyperosmotic adaptation	[86]
*S. cerevisiae*	HOG MAPK pathway	HOG signaling coordinates glycerol accumulation and gene expression during desiccation stress	[87]
*S. cerevisiae*	Energy metabolism	More tolerant strains maintain higher mitochondrial activity and ATP during drying & rehydration	[88]
Yeast deletion collection	Functional genomics	Genome-wide deletion library identifies genes required for survival under various stress conditions	[89]
*S. cerevisiae*	Structural changes during desiccation	Desiccation causes loss of vacuoles, lipid droplets, mitochondrial cristae	[90]
*D. hansenii*	Polyol and carbohydrate accumulation	Under desiccation, accumulates glycerol and trehalose resulting in polyols & sugars act as paired Osmo protectants	[91]
*S. cerevisiae*	Cell cycle & chromatin	Chromatin compaction in G1 phase is linked to increased desiccation tolerance	[92]
Proteomic analysis	Protein stability	Proteins enriched in charged and hydrophilic residues resist aggregation during drying	[93]
Yeast expressing tardigrade CAHS proteins	Combined protection with trehalose	CAHS proteins, particularly with trehalose, enhance desiccation tolerance	[94]

Further molecular research on this topic offers the opportunity to gain new insights into the function and role of pathways that protect yeast cells from osmotic and dehydration stress. According to data presented by Lee et al. [33], the stress-specific functions of the glycerol-3-phosphate dehydrogenases Gpd1 and Gpd2 are determined by their phosphorylation states. The *GPD1* gene plays a crucial role in the hyperosmotic adaptation of yeast cells. Hohmann [87] elucidated the mechanism by which the HOG MAPK pathway coordinates these biochemical responses by regulating glycerol content. Another factor determining stress conditions is the availability of energy. According to data presented by Kuliešienė et al. [88], cells that are more resistant to dehydration exhibit increased mitochondrial activity and higher ATP levels. Furthermore, the authors concluded that adequate maintenance of the basal energy reserve by yeast is essential for subsequent cell development and regeneration. Functional genomics studies have significantly expanded our understanding the desiccation tolerance across various yeast species. According to Giaever and Nislow [89], increasing deletion mutations in yeast cells enables the identification of genes essential for survival under different stress conditions.

Biotechnological research on microbial genomics has significantly expanded our understanding of microbial adaptation to dehydration. According to Giaever and Nislow, maintaining a collection of individual yeast strains that are mutants (with knockout genes) allows us to identify genes essential for survival under various stress conditions. Furthermore, Ren et al. [90] used microscopy and genetic/biochemical manipulations to demonstrate that *S. cerevisiae* undergoes significant structural changes during desiccation, including loss of vacuoles, lipid droplets, and mitochondrial cristae. The authors linked desiccation tolerance to lipid metabolism. Moreover, the existing literature confirms that yeast survival depends not only on chaperones and signaling pathways, but also on the activity and changes in the architecture of the wall-membrane complex and on cell metabolism.

Studies of unconventional yeasts highlight an even greater diversity of microbial survival strategies. Gonzalez-Hernandez et al. [91] demonstrated that under salt stress and drying conditions, the halotolerant *Debaryomyces hansenii* accumulates high levels of glycerol and trehalose, indicating that polyols and carbohydrates act as a pair of osmo protectants. Structural changes also enhance yeast’s ability to survive harsh environmental conditions. Sęk et al. [11] reported that modifications, such as changes in the lipid composition of the cell membrane or cell wall stiffness, help limit cellular damage during dehydration. According to Zhang and Zhang [92], cells in the G1 phase, which are characterized by compact chromatin, exhibit significantly increased tolerance, suggesting that genomic organization plays a role in stress resistance. Proteomic studies by Romero-Pérez et al. [93] demonstrated that proteins characterized by a high number of hydrophilic residues on their surfaces are less susceptible to aggregation upon drying. Nguyen et al. [94] found that expression of tardigrade CAHS proteins in yeast increased their tolerance to stress conditions.

The literature presented here indicates that yeast employs a complex set of responses, along with structural and metabolic mechanisms, to survive dehydration. Furthermore, yeast cells can resume their physiological activity after rehydration. This survival is achieved through a combination of stress-related signaling pathways, the accumulation of protective substances, chromatin remodeling, and membrane restructuring.

## Genetic Determinants of Anhydrobiosis in Yeast

**Fig. 2:**
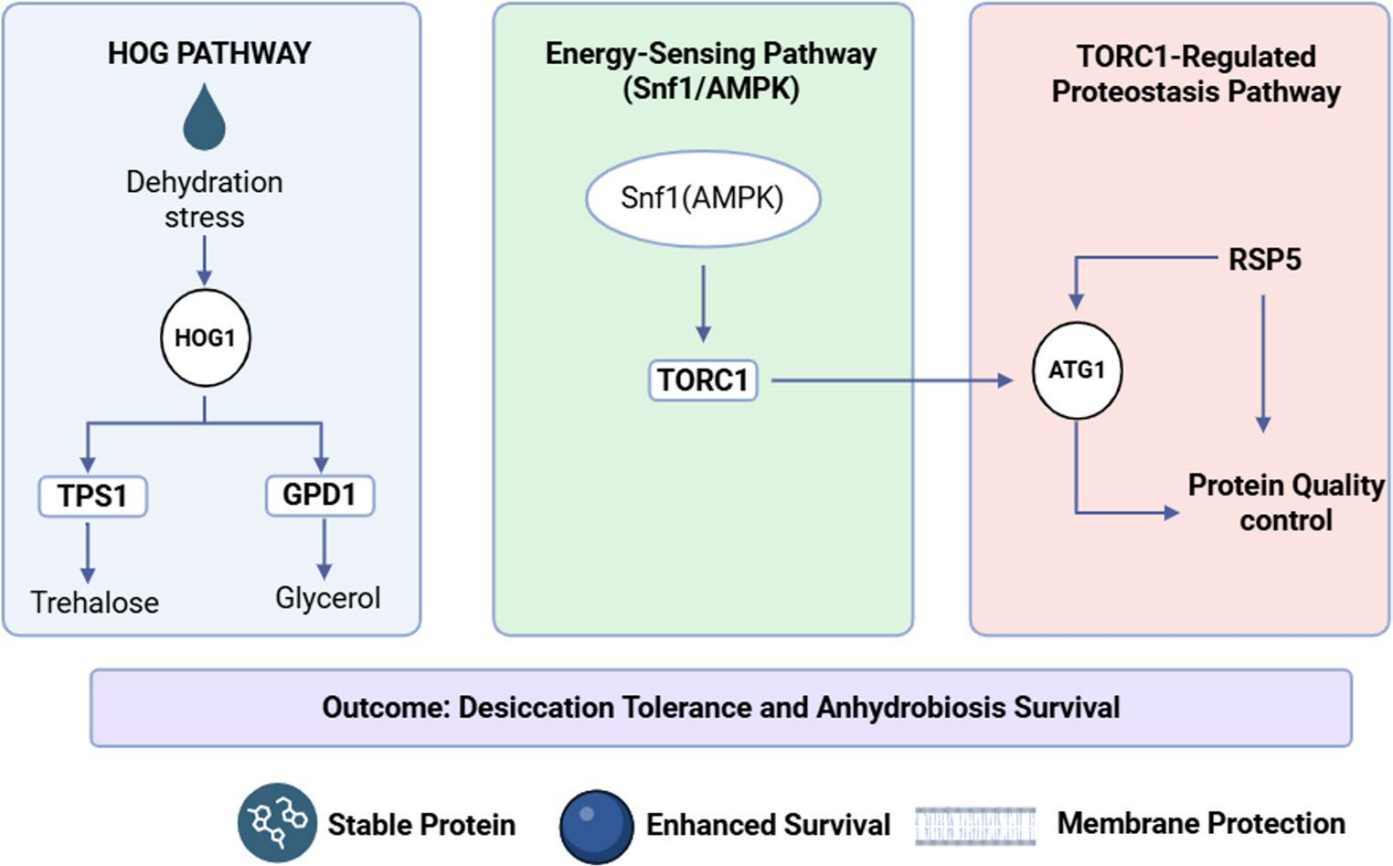
Molecular pathways regulating desiccation tolerance in yeast during anhydrobiosis. Figure was prepared with BioRender online software (www.Biorender.com)

Yeast can survive unfavorable dehydration conditions by coordinating stress signals, altering its metabolism, and degrading proteins. Furthermore, yeast can activate a set of genes essential for proper cellular function under extreme dehydration conditions. The presented molecular mechanisms influence the activity of key signaling pathways, including the high-osmolarity glycerol (HOG) process pathway, the Snf1/AMPK energy pathway, and the TORC1 signaling pathway (Fig. 2).

### Molecular Pathways Involved in Dehydration Response

The HOG pathway is a key element in the cellular response to dehydration-related stress. Hog1, through phosphorylation of the transcription factors Msn2 and Msn4, increases the expression of genes involved in Osmo protection and autophagy mechanisms [24]. These processes result in increased synthesis of trehalose and glycerol. Moreover, trehalose, together with glycogen, helps maintain cellular osmotic balance under dehydration conditions [95, 96]. Meanwhile, energy-sensing kinase Snf1 (yeast AMPK ortholog) is sensitive to nutrient levels and phosphorylates Msn2/4 and inhibits TORC1, which facilitates the induction of autophagy and transition to metabolic quiescence [49, 97–99]. In the case of autophagy, the primary conserved genes are ATG1, which triggers the formation of autophagosomes, and ATG8, which facilitates membrane expansion. At the same time, the E3 ubiquitin ligase Rsp5 tags damaged proteins for selective degradation via K63-linked polyubiquitination [100–103]. Functional studies revealed that the absence of HOG1, MSN2/4, TPS1, or RSP5 results in a complete loss of desiccation tolerance, indicating that these genes are critical for maintaining cellular health and proteostasis under anhydrobiosis conditions [82, 104]. The interplay between individual HOG and Snf1 pathways is also essential, as they enhance cell response to changing culture conditions induced by stress factors. Yeast cells adjust their metabolism in response to osmotic stress, enabling efficient trehalose and glycerol biosynthesis even under conditions of nutrient deficiency [105, 106].

Moreover, the mitochondrial retrograde response (controlled by Rtg1/3) is triggered during dehydration, signals mitochondrial stress to the nucleus and modulates amino acid synthesis and NAD^+^ homeostasis. A study by Di Noia et al. [107] showed that when HAP4, a factor regulating mitochondrial function, was disabled, yeast adapted more rapidly to osmotic stress via an RTG2-dependent pathway. This demonstrates that mitochondrial metabolic state plays a vital role in stress adaptation.The combination of these genetic circuits enables yeast to rapidly transition between metabolic activity and dormancy, and vice versa, during rehydration.

## Epigenetic Modifications and Stress Memory

Hog1, as a key element in chromatin remodeling processes, facilitates changes in nucleosome structure, which in turn enables the initiation of transcription [108]. Simultaneously, epigenetic changes, including histone methylation and acetylation can be used to create stress memory and enable cells to react more effectively to subsequent dehydration [27] (Fig. 2). Gene promoters that were initially stimulated by stress are also marked by the methylation of histone H3K4 mediated by the Set1/COMPASS complex, resulting in quicker transcriptional re-induction on recurring exposure to stress [22, 109, 110]. In addition, Hog1 interacts directly with the RSC (Remodels the Structure of Chromatin) complex to reposition nucleosomes at stress promoters, such as GPD1 and CTT1, thereby ensuring rapid recruitment of RNA polymerase II [111]. The interaction between MAPK signalling and chromatin modifiers is a complex process that connects signal transduction and epigenetic regulation during anhydrobiosis [112]. Genome-wide ChIP-seq studies have shown that dehydration stress leads to the temporary redistribution of histone marks [113], such as an increase in H3K9ac and H3K14ac at stress-responsive gene promoters, which is partially reversed with rehydration [112]. These reversible modifications are accompanied by dynamic changes in the occupancy of chromatin remodelers such as SWI/SNF and INO80, indicating extensive nucleosome reorganization during recovery [114]. Other forms of regulation include noncoding RNAs (ncRNAs), which are transcribed by stress-responsive promoters to facilitate recruitment of chromatin modifiers and transcription factors during reactivation phases [113, 115]. Components of the Set3 histone deacetylase complex also stabilize the transcriptional stress memory by stabilizing specific chromatin accessibility states, which facilitate faster gene induction on the subsequent stress of osmotic or dehydration [113]. Interestingly, the transcriptional advantage of repeated dehydration-rehydration cycles in cells is heritable, even after many generations of growth under standard conditions. This implies that some chromatin marks, or their functional implications, are maintained across mitosis and lead to long-term adaptation [116]. Higher eukaryotes have been shown to exhibit similar memory effects, whereby yeast anhydrobiosis has been linked to conserved cellular resilience and aging control mechanisms [27].

## Transcriptomic and Chromatin Accessibility Studies

In recent years, research using biotechnological methods (e.g., transcriptome studies) has facilitated the interpretation of the impact of various stress conditions on yeast cell function. Examples of such analyses include RNA sequencing (RNA-seq) and chromatin accessibility analysis using ATAC-seq. These research techniques are attracting increasing interest from various research centers worldwide. Obtaining results using these methods can yield extensive insights into the hierarchical control of gene expression and chromatin remodeling during anhydrobiosis.

In transcriptomic studies, hundreds of genes are reported to undergo expression changes in dehydration/desiccation, with enrichment in trehalose metabolism (e.g., TPS1), ROS defense genes (e.g., *SOD2*, *CTA1*), and autophagy genes (e.g., *ATG8*, *ATG32*) [84, 117, 118]. ATAC/MNase-based and chromatin profiling show that a greater amount of accessibility and nucleosome remodelling of the stress promoters are observed with the presence of STRE/HSE motifs and Msn2/4/Hsf1 activity [119–121]. The integration of molecular mechanisms, exemplified by transcriptome and epigenome data in yeast cells, is a response to changing culture conditions driven by various stress factors. Furthermore, active genetic promoters in the yeast cytosol remain in a state of chromatin readiness, enabling rapid transcriptional reactivation during rehydration [122–124]. Studies of various regulatory processes occurring in the yeast cell cytosol have revealed characteristic gene expression patterns that link anhydrobiosis mechanisms with the desiccation resistance observed in nematodes and tardigrades. The literature data presented here demonstrate that so-called stress memory and the activation of mechanisms controlling protein stability are essential evolutionary elements that influence the survival strategies of various organisms.

## Potential Parallels to Neuroprotective Mechanisms

The molecular mechanisms of survival in yeast under anhydrobiosis show conceptual convergence with pathways implicated in neurodegeneration, making desiccation tolerance a useful comparative framework for exploring neuroprotective mechanisms [125]. A central aspect of this phenomenon is the alteration of proteostasis. Such phenomena can be observed in Alzheimer’s disease (AD), Parkinson’s disease (PD), and Huntington’s disease [126]. Experimental research has shown that yeast disaggregase Hsp can be modified to dissolve amyloid aggregates associated with human neurodegenerative diseases [127]. Hsp104 and its homologs engineered variants rescue the proteostasis of amyotrophic lateral sclerosis (ALS) and Parkinson’s disease (PD) models by reactivating alpha-synuclein and TDP-43 inclusions [128]. These results underscore the utility of yeast derived chaperone systems as experimental tools for investigating protein aggregation rather than implying physiological equivalence between yeast and neuronal systems.

Significantly, trehalose, a major chemical chaperone in yeast anhydrobiosis, inhibits the aggregation of neurodegeneration-related proteins in vitro and in animal models [32]. Importantly vertebrates do not synthesize trehalose therefore, the observed effects in animal models result from exogenously administered trehalose [129]. Trehalose suppresses α-synuclein oligomerization in PD mice and improves motor deficits by stabilizing proteasome activity and increasing autophagy [130]. In Huntington’s disease models, trehalose has been reported to limit mutant huntingtin aggregation and to improve cellular stress resilience, potentially through enhanced autophagy and mitochondrial homeostasis [131–133]. Furthermore, the presented information highlights the neuroprotective potential of this non-reducing glucose disaccharide [134].

Studies of biomolecular condensates formed during yeast dehydration—such as chaperone-based structures containing intrinsically disordered proteins (IDPs) show apparent similarities to condensates observed in higher organisms [67]. These structures are dynamic and arise from liquid-liquid phase separation. However, it should be emphasized that these similarities stem from universal biophysical principles, not complete functional equivalence between species. Unlike neuronal stress granules (SGs) observed in ALS/FTD, yeast condensates serve a protective function-temporarily and reversibly binding misfolded proteins to prevent their aggregation. In neurons, however, similar structures can become fixed over time and form pathological aggregates, especially in the presence of mutations in TDP-43 or FUS [135, 136]. Trehalose also plays a significant role in modulating the viscosity of yeast condensates. This mechanism could provide a model for designing compounds that stabilize stress granules [137]. These comparisons help us understand the general mechanisms underlying stress-induced maintenance of phase structure, although it should be remembered that pathological aggregation in neurons is much more complex [138–140]. In yeast, K63-type ubiquitination (involving RSP5) and ATG1 activation initiate selective autophagy. These mechanisms are evolutionarily conserved [141]. It is worth noting that AMPK activators and mTOR pathway inhibitors, such as metformin or resveratrol, enhance the autophagy process. This effect is observed in both yeast cells and neurons, supporting the maintenance of proteostasis [142, 143]. Yeast anhydrobiosis is a simplified experimental model of the cellular response to stress, based on evolutionarily conserved biological mechanisms [144, 145], and is not a direct model of neurodegenerative diseases or aging processes.

## Mechanisms of Anhydrobiosis and New Strategies to Counteract Neurodegenerative Diseases

Anhydrobiosis is an interesting model for studying the adaptation of various cells to extreme environmental (stress) conditions and their long-term viability [11]. The mechanisms that ensure yeast survival during dehydration, including autophagy and proteostasis, are directly linked to the processes that determine their lifespan [146]. Studies of the pathways that maintain the structural integrity of yeast cells may provide conceptual insights into neurodegenerative disorders characterized by protein aggregation and metabolic abnormalities. However, this does not mean that they will offer a direct model for translational applications [147, 148]. In anhydrobiosis, yeast cells enter a state of metabolic dormancy while retaining the ability to return to full physiological activity quickly. The transition requires the activation of the HOG-MAPK cascade, TORC1 inhibition, and selective autophagy mediated by Atg1 and Atg13 signaling [149–151]. The balance between protein folding (Hsp70, Hsp104) and degradation (proteasome, autophagy) determines the point at which survival and cell death occur [152–154]. Such regulatory balance mirrors neuronal responses to misfolded proteins, where loss of autophagy or chaperone function leads to synaptic dysfunction and neurodegeneration [155, 156]. The metabolism of trehalose is one of the most promising interconnections between neuronal protection and yeast anhydrobiosis [33]. In yeast, trehalose maintains membranes and proteins during desiccation and provides energy during recovery after rehydration [83]. Trehalose also induces autophagy in mammalian cells, independent of mTOR, and inhibits the aggregation of toxic proteins, such as α-synuclein and huntingtin [157]. The other important mechanism is the regulation of phase-separated condensates. Yeast utilizes IDP chaperone complexes to store unfolded proteins. This process prevents irreversible amyloid formation [158, 159]. Furthermore, stress granules containing TDP-43 or FUS proteins exhibit defective phase transitions in neuronal cells, which may facilitate amyloid fibril aggregation [160]. Understanding how yeast cells maintain fluidity and physiological activity, in part through trehalose and Hsp12, may aid further investigation into the general principles of proteome stabilization under stress conditions [161, 162]. Pharmacological methods based on yeast research include TORC1 inhibitors (rapamycin, everolimus) and AMPK activators (metformin, resveratrol), which can be used to induce autophagy and decrease the load of aggregates [163, 164]. The use of engineered disaggregates, which are yeast Hsp104 scaffolds, is also under investigation for their capacity to reactivate misfolded neuronal proteins [165]. It’s important to emphasize that stress responses are more complex in multicellular organisms. Desiccation tolerance may represent a general approach to coping with stress related to protein damage and metabolic disruption. The HOG/MAPK, AMPK, and TORC1 conserved signaling networks are the master regulators of environmental stress responses in yeast and neuroprotective pathways in mammals [166, 167]. Future research should use a combination of yeast genetics, structural biology, and drug screening to investigate compounds that replicate the protective effects observed during anhydrobiosis in yeast [168].

Therefore, the mechanisms discovered in yeast during anhydrobiosis offer new perspectives for conceptually informing approaches to combat age-related neurodegenerative diseases. Understanding the processes of proteome stability and metabolic homeostasis in simple cells subjected to adverse conditions can guide the development of treatments to maintain neuronal activity, slow the progression of aging, and make cells more resilient to stress.

## Applications of Anhydrobiosis in Biotechnology and Industry

**Fig. 3:**
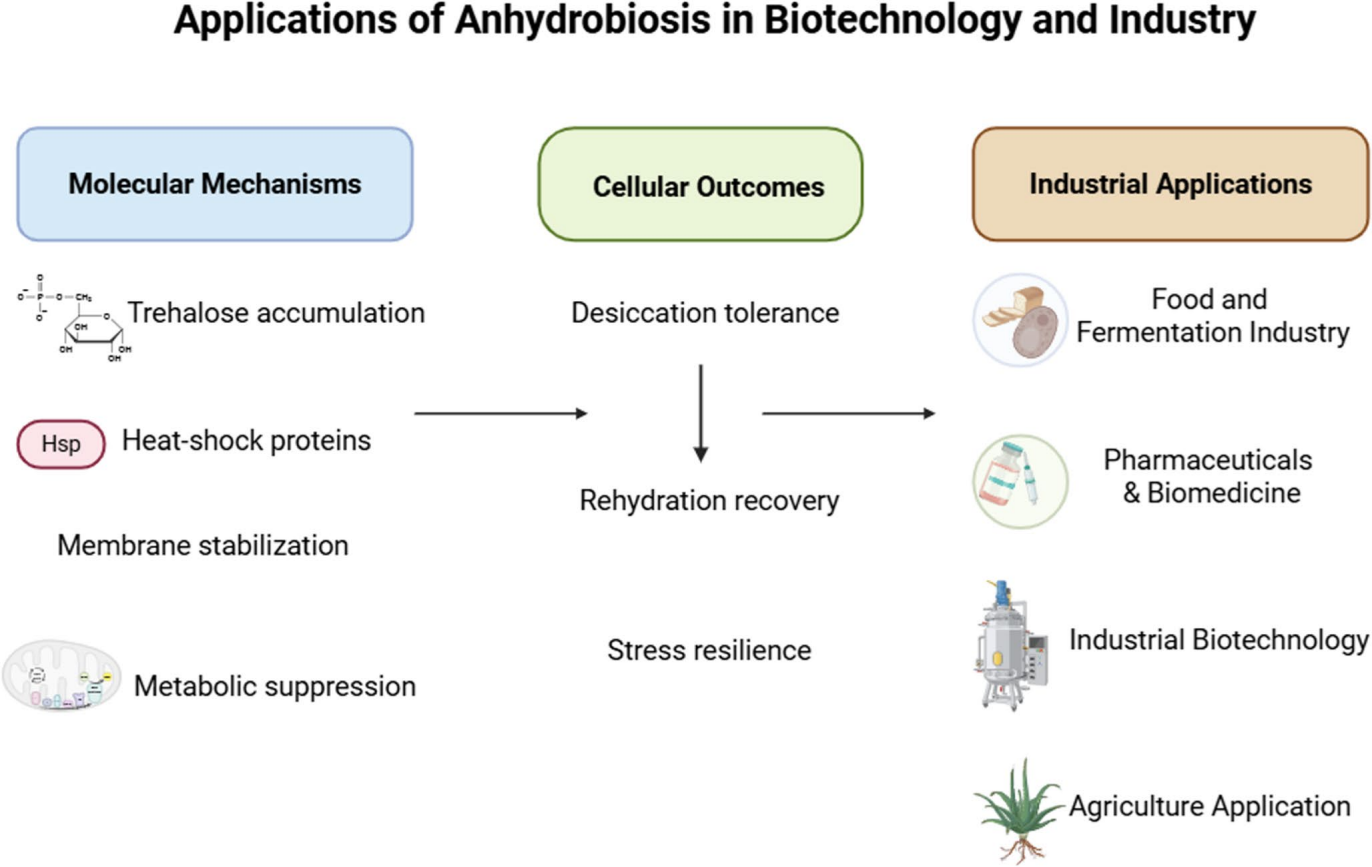
Presentation of molecular mechanisms (accumulation of trehalose, heat shock proteins, cell membrane stabilization, and metabolic suppression) that influence yeast cell tolerance to desiccation and stress resistance. These properties are utilized in various industries, including food production, pharmaceuticals, and agriculture. Figure was prepared with BioRender online software (www.Biorender.com)

Anhydrobiosis is now a significant biological term widely used in biotechnology, agriculture, and industry (Fig. 3). The remarkable ability of cells to endure nearly complete dehydration through molecular stabilization and metabolic suppression has prompted the development of methods to enhance biological preservation, stress resistance, and product stability. The traditional yeast, including *Saccharomyces cerevisiae*, and non-traditional yeasts, such as *D. hansenii*, *Yarrowia lipolytica*, and *Pichia kudriavzevii*, offer systems with diverse applications in the study and exploitation of anhydrobiotic processes. Anhydrobiosis underpins active dry yeast (ADY) technology in the food and fermentation industries, one of the most popular biotechnological products [169]. Industrial strains of *S. cerevisiae* are dried in a manner that simulates natural desiccation to maintain metabolic activity and fermentation performance during long-term storage [170]. Stress-response systems are also similar in *D. hansenii* and *P. kudriavzevii*, which can withstand higher osmotic pressure and are increasingly used in baking, wine fermentation, and the manufacture of fermented foods under extreme conditions [171, 172].

The molecular understanding of yeast anhydrobiosis has been applied in the pharmaceutical and biomedical industries to develop dry-preservation methods for vaccines, enzymes, and therapeutic proteins [169, 173–175]. The glass-forming sugars, such as trehalose, form stable amorphous matrices that maintain biological activity even without the presence of water [176]. These ideas have facilitated the development of thermostable vaccine preparations that do not require cold-chain storage [177]. In addition, the expression of yeast stress-protective genes in bacterial or mammalian cells enhances their desiccation tolerance, providing new opportunities for synthetic biology and biomedical applications. Both traditional and non-traditional yeasts used in industrial biotechnology and biofuel production exhibit natural or artificial tolerance to ethanol, osmotic stress, and dehydration [178]. Oleaginous yeasts such as *Y. lipolytica* are being considered in the production of biofuels and lipids due to their excellent stress resistance and rapid recovery after drying [179]. Bioprocesses can be made more sustainable through the development of dried biocatalyst formulations that are easy to store and reuse, thereby reducing energy consumption and enhancing sustainability [180, 181].

Anhydrobiosis-based methods are also being applied in the agricultural sector. Desiccation-tolerant yeasts and fungi are bioinoculants that enhance the growth and resistance of plants to drought or heat stress [182]. Formulations containing anhydrobiotically preserved microbial spores have a long shelf life and high field performance, which improves soil fertility and crop yields [183]. Studies on bacterial anhydrobiosis have demonstrated that spray-drying and freeze-drying can be used to preserve microorganisms as biofertilizers, plant-growth-promoting agents, and biocontrol microbes, providing a prototype for developing anhydrobiotically preserved yeast preparations for agricultural use [184]. Protective compounds, such as trehalose and mannitol, produced by yeast, are also being studied as bio-stimulants to enhance drought resistance in plants [185].

The increasing understanding of the molecular mechanisms of desiccation resistance across various yeast species will enable future application of this knowledge in the design of biotechnological processes and the creation of stable, stress-resistant bioproducts. These solutions have potential applications in numerous scientific fields, including food technology, agriculture, and environmental sciences.

## Prospects for Future Research

### Metabolism, Mitochondrial Signaling, and Nutrient Sensing

To further explore the concept of anhydrobiosis as a model for aging and longevity, future studies in yeast should integrate research on autophagy, metabolism, and stress-response pathways. Furthermore, analysis of xerotolerant yeasts (*D. hansenii*,* Y. lipolytica*, and *Candida parapsilosis*) may be necessary for understanding novel adaptive strategies in lipid remodeling, osmolyte production, and mitochondrial protection. These mechanisms could provide a solid starting point for further discoveries regarding long-term cell survival [186–189]. The mechanisms by which trehalose metabolism, mitochondrial dynamics, and nutrient-sensing pathways, including the TOR and AMPK pathways, can be utilized to survive extreme dehydration should be studied [190–193]. Past research suggests that dehydration and caloric restriction are both associated with changes in redox balance and energy metabolism; however, the mechanisms by which these processes change during drying and rehydration cycles remain unclear [19, 194]. The temporal dynamics of these pathways should be investigated in future experiments to determine whether dormancy triggers a caloric restriction-like metabolic state [195].

### Stress Memory and Long-Term Cellular Survival

Research conducted in various centers worldwide has increased understanding of the functioning of regulatory mechanisms and epigenetic markers associated with the long-term survival of yeast cells subjected to anhydrobiosis [196–198]. This progress is possible thanks to the development of techniques such as transcriptomics, proteomics, metabolomics, and analysis of chromatin accessibility. Integrating RNA-seq and ATAC-seq with single-cell technologies will enable the identification of heterogeneity within the population in responses to stress and the revelation of subpopulations with extraordinary desiccation resistance [199, 200].

### Proteostasis, Post-Translational Regulation, and Translational Perspectives

A combination of phosphoproteomics and acetylomics can also be used to identify post-translational switches in autophagy and proteostasis. In addition, CRISPR-based gene editing and live-cell imaging provide powerful tools to study how autophagy and metabolic changes occur during the dehydration and rehydration cycle in anhydrobiotic yeast [201]. Dynamic processes in the cell can now be monitored in real time using fluorescent reporters that signal, among other things, Atg8 lipidation, changes in mitochondrial membrane potential, and ROS levels [202]. Genome-wide screens using CRISPR technology enable the identification of unknown genes and non-coding RNAs. These genetic factors in yeast cells coordinate transcriptional and metabolic processes in response to stress conditions [203]. These insights should also be translated into cross-kingdom applications in the future. Synthetic-biology methods may be applied to recreate the conditions of anhydrobiosis in mammalian or plant cells by expressing genes that help overcome stress in yeast, such as trehalose synthase (TPS1) or Hsp12 [19, 204]. Computational studies of the stress-response pathways in yeast can also be used to estimate how the regulation of TORC1, AMPK, and HOG signals influences cell survival [205]. Expanding knowledge in this field may support the development of therapies that enhance proteostasis and improve resistance of aging tissues to chronic stress [206, 207]. The long-term combination of molecular biology, omics technologies, and computational modeling will provide a better understanding of cellular lifespan extension and resilience.

## Conclusion

The process of anhydrobiosis occurring in yeast cells is an example of a complex mechanism. Yeast cells have evolved various mechanisms to adapt to stressful conditions through protein quality control (proteostasis), autophagy, metabolic regulation, and chromatin remodeling. All of these aspects help maintain cell integrity during severe dehydration. Numerous studies have demonstrated that a single signaling pathway does not control desiccation tolerance. It results from the combined actions of trehalose accumulation, HOG-MAPK signaling, and mitochondrial energy maintenance. The presented processes not only discuss the possibilities for yeast cell survival under extreme drought conditions but also provide insight into the functioning of individual mechanisms of cellular resistance to stress factors and longevity. A growing knowledge of yeast anhydrobiosis is now directly influencing practical areas, such as the stabilization of biological products, the engineering of stress-resistant industrial strains, and the development of stable microbial inoculants for agricultural use. Further study of genetic diversity in both traditional and non-traditional yeasts will likely reveal additional protective mechanisms that can be utilized in biotechnology and provide valuable insights into studying stress responses in more complex eukaryotes.

## Data Availability

No datasets were generated or analysed during the current study.
